# A Life-Threatening Incidence of Neurotoxic Indian Krait Snake Bite: A Case Report

**DOI:** 10.7759/cureus.27719

**Published:** 2022-08-05

**Authors:** Tejaswee Lohakare, Bibin Kurian, Archana Maurya, Mayur B Wanjari, Khushbu M Meshram

**Affiliations:** 1 Child Health Nursing, Smt. Radhikabai Meghe Memorial College of Nursing, Datta Meghe Institute of Medical Science (DU), Wardha, IND; 2 Research and Development, Jawaharlal Nehru Medical College, Datta Meghe Institute of Medical Sciences (DU), Wardha, IND

**Keywords:** paralysis, unconscious, anti-convulsion, snakebite, neurotoxins, indian krait

## Abstract

The Indian Krait delivers one of the most lethal venoms compared to other Asian snakes. The venom of the common Krait comprises substantial neurotoxins that cause muscular paralysis. Significant snake bite incidence occurs in rural areas. The significant death rate caused by snake bites is seldomly reported in the medical literature. A 14-year-old adolescent girl was brought by her parents to the emergency department (ED) in an unconscious state. The patient reported swelling on her right hand with fang marks of a snake bite, sweating, and increased salivation. The primary therapeutic intervention was given to the patient and she was treated with intravenous anti-snake venom serum, antibiotics, and anti-epileptics during hospitalisation.

## Introduction

Kraits usually bite at night when they enter dwellings in search of food [1]. After neurotoxic envenomation caused by a common Krait bite, the patient requires a very large dose of polyvalent anti-snake venom (ASV) to overcome the neurological manifestations [2]. All of the patients were from impoverished agricultural families living in villages, and the vast majority of them (96%) slept on the floor. The majority of the bites occurred at night as the victims slept on the floor [3].

High mortality from toxic snakebite is a serious health issue. It is a source of concern for medical professionals. Clinically, snakebite envenomation is divided into two categories: neurotoxic and vasculotoxic. Cobra and Krait are both neurotoxic. It is primarily composed of a highly strong presynaptic neurotoxin that prevents impulses from nerve terminals from being transferred to muscle receptors. Although the venom contains a few additional neurotoxic ingredients, it has no cytotoxic, haematotoxic, or other components [4]. Case fatality rates can be higher when patients do not have immediate access to life-saving anti-snake venom serum (ASVS), which is frequent in developing-country rural communities [5].

## Case presentation

A 14-year-old adolescent girl was brought by her parents to the ED in an unconscious state with a complaint of a snake bite during the day. Primary preventive measures were taken by the physician. Her parents stated that she was in her usual state of health until the afternoon when suddenly they found their child unresponsive in the house and they noticed a bite mark on her right-hand finger with discolor. The parents primarily visited the local area physician and he revealed a snake bite based on the physical examination and referred her to the multispecialty hospital.

**Figure 1:**
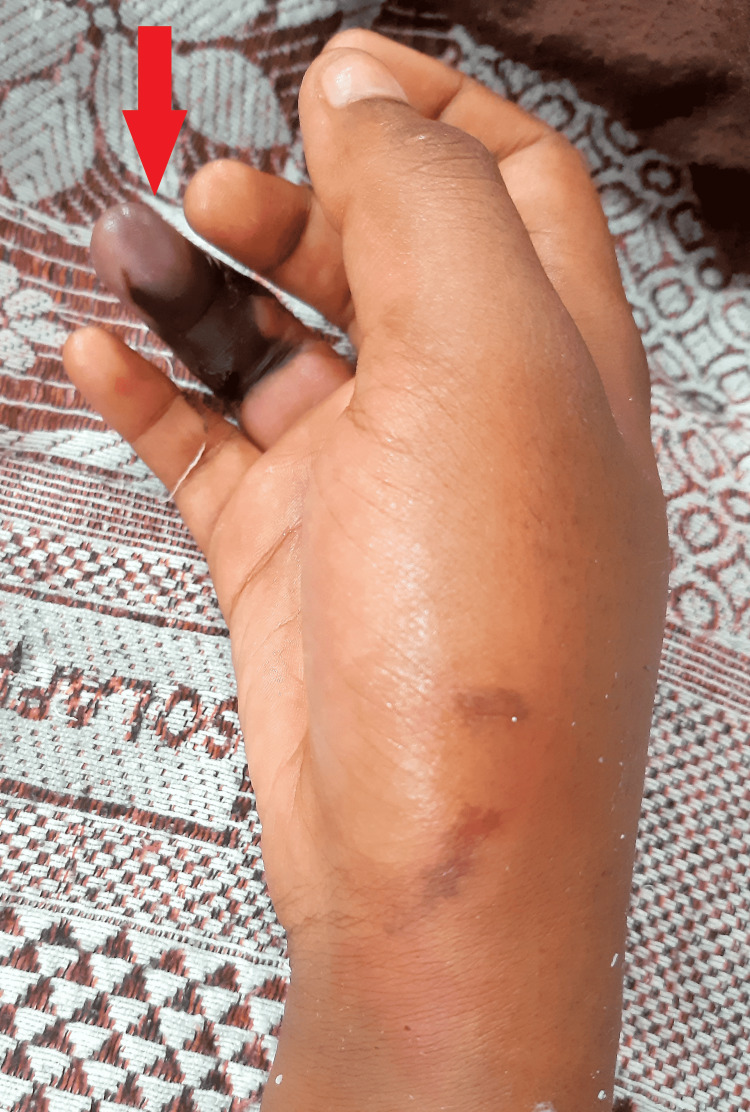
Clinical image shows gangrene formation on the ring finger of right hand at site of the Krait snake bite.

On physical examination, swelling on her right hand with a fang mark, sweating, bradycardic, bradypnea, and salivation were observed. Vital signs included heart rate 30 bpm and were essentially nearing full cardiac arrest with unrecordable other vital signs. Immediately Pediatric Advanced Life Support (PALS) was started. After completion of three rounds of PALS, the carotid pulse was palpable and the patient reverted to spontaneous circulation (ROSC). The patient was moved to the pediatric intensive care unit (PICU) for further management (Figure 1).

Upon arrival at the PICU, the patient was endotracheally intubated. Simultaneously the patient was administered intravenous snake venom antiserum in 20 vials, diluted with 10 ml NS in each vial, and administered 200 ml over 30 minutes. On laboratory investigation, complete blood count and renal function were all within normal range. On the fourth day of hospitalization patient's vitals were stable. Medical management continued and patient prognosis was good.

## Discussion

In modern India, snakebite remains an underestimated cause of accidental death. The death due to snake bite is 40 to 50 thousand per year, and most of the deaths occur in rural areas due to the poor availability of the health care system [6]. Many superstitions and myths regarding snake bites cause a delay in receiving emergency treatment modalities to the patient. The neurotoxic snake bite is significantly associated with a high death rate due to immediate respiratory failure, mostly in rural areas [7].

Many patients with snake bites are treated and die outside medical facilities, especially in rural India [8]. The burden of snake bites is similar to infectious disease because many people in rural areas have died over the years. For example, there is one death from snake bite for every two deaths from HIV in India. Moreover, there is a need for education and awareness programs about snake bites in rural and urban areas that can prevent death [9].

Management of the Krait snake bite clinical manifestation protocol should be provisioned. A patient with a snake bite requires proper ventilation, primary emergency management, and maintaining a normal blood pressure range; these can all improve the patient's prognosis and mortality [10]. In the present case, medical management was received on time, and antivenom therapeutic intervention was given to the patient. The patient's prognosis was good, and now she maintains her vital signs and is conscious to the time, place, and person.

## Conclusions

In India, mortality due to the Krait snake is more prevalent in rural areas due to the lack of awareness and education regarding the snake bite. There is a need for education and awareness programs for the rural people to be aware of and understand the importance of hospitalization. Mostly rural people were treated first in the village by the local practitioner or the local person. There is a need for education to the people regarding primary treatment having to be given as early as possible to reduce the systemic poisoning and life-threatening symptoms. In this case, the patient lived near the hospital area, and her parents brought her immediately to the hospital. The patient got a standard line of treatment during the golden period. Because of that, her life was saved, and the patient’s prognosis was good. 
